# Liraglutide downregulates hepatic LDL receptor and PCSK9 expression in HepG2 cells and db/db mice through a HNF-1a dependent mechanism

**DOI:** 10.1186/s12933-018-0689-9

**Published:** 2018-04-04

**Authors:** Sheng-Hua Yang, Rui-Xia Xu, Chuan-Jue Cui, Yin Wang, Ying Du, Zhi-Guo Chen, Yu-Hong Yao, Chun-Yan Ma, Cheng-Gang Zhu, Yuan-Lin Guo, Na-Qiong Wu, Jing Sun, Bu-Xing Chen, Jian-Jun Li

**Affiliations:** 1 Division of Dyslipidemia, State Key Laboratory of Cardiovascular Disease, Fuwai Hospital, National Center for Cardiovascular Diseases, Chinese Academy of Medical Sciences, Peking Union Medical College, BeiLiShi Road 167, Beijing, 100037 China; 2 Department of Cardiology, Beijing Tiantan Hospital, Capital Medical University, Beijing, 100050 China

**Keywords:** PCSK9, Low-density lipoprotein receptor, Glucagon-like peptide-1, Liraglutide, Type 2 diabetes mellitus

## Abstract

**Background:**

Proprotein convertase subtilisin/kexin type 9 (PCSK9), a major regulator of cholesterol homeostasis, is associated with glucose metabolism. Liraglutide, a glucagon-like peptide-1 receptor agonist, can increase insulin secretion in a glucose-dependent manner and lower blood glucose. We aimed to investigate the relationship between liraglutide and PCSK9.

**Methods:**

At the cellular level, the expressions of PCSK9 and hepatocyte nuclear factor 1 alpha (HNF1α) protein in HepG2 cells stimulated by liraglutide was examined using Western blot. Seven-week old db/db mice and wild type (WT) mice were administered either liraglutide (200 μg/kg) or equivoluminal saline subcutaneously, twice daily for 7 weeks. Fasting glucose level, food intake and body weight were measured every week. After the 7-week treatment, the blood was collected for lipid and PCSK9 levels detection and the liver was removed from the mice for oil red O staining, immunohistochemical analysis, immunofluorescence test and Western bolt.

**Results:**

Firstly, liraglutide suppressed both PCSK9 and HNF1α expression in HepG2 cells in a time and concentration dependent manner. Secondly, liraglutide induced weight loss in WT and db/db mice, decreased serum PCSK9, glucose and lipid levels and improved hepatic accumulation in db/db but not WT mice. Thirdly, liraglutide reduced both hepatic PCSK9 and low-density lipoprotein receptor (LDLR) expression with a decrease in HNF1α in db/db mice but not in WT mice.

**Conclusions:**

Liraglutide suppressed PCSK9 expression through HNF1α-dependent mechanism in HepG2 cells and db/db mice, and decreased LDLR possibly via PCSK9-independent pathways in db/db mice.

**Electronic supplementary material:**

The online version of this article (10.1186/s12933-018-0689-9) contains supplementary material, which is available to authorized users.

## Background

Proprotein convertase subtilisin/kexin type 9 (PCSK9), mainly secreted by the liver as an important regulator of cholesterol homeostasis, enhances the endosomal and lysosomal degradation of hepatic low-density lipoprotein (LDL) receptors (LDLR), resulting in increased circulating LDL-cholesterol (LDL-C) concentration [1, 2]. Interestingly, recent data suggested a relation of PCSK9 to glucose metabolism [3–11]. The epidemiological studies previously revealed positive associations between plasma PCSK9 and fasting glucose and insulin [3, 10–12]. Several studies have suggested that an increase in PCSK9 levels is associated with higher fasting insulin levels in general populations and patients with diabetes [3, 10, 11]. Conversely, our previous studies and others found no significant difference in PCSK9 levels between the patients with or without diabetes [4, 9]. Awan et al. reported that presence of the loss-of-function PCSK9 p.R46L mutation was associated with insulin resistance in subjects with apolipoprotein E3/E2 genotype [13]. Furthermore, clinical trials showed that PCSK9 inhibitors did not increase the risk of onset-diabetes [14, 15], whereas genetics studies exhibited a link between PCSK9 genetic variants and risk of type 2 diabetes [16, 17]. To date, however, the exact mechanism of PCSK9 involved in glucose metabolism is still undetermined.

Liraglutide, one of glucagon-like peptide-1 (GLP-1) receptor agonists, can stimulate glucose-dependent insulin secretion, suppress glucagon release, and reduce food intake, resulting in glycemic improvement and weight loss in patients with type 2 diabetes [18–22]. In addition of anti-diabetic and antiobesity effect, liraglutide can reduce cardiovascular events [23], and thus has been widely used for the treatment of type 2 diabetes. Until now, however, no data concerning the role of liraglutide in PCSK9 levels are available. Therefore, in the present study, the impact of liraglutide on PCSK9 expression was assessed in HepG2 cells and mice.

## Materials and methods

### Cell culture and treatment

The human hepatoma cell line, HepG2, was obtained from Cell Resource Center, IBMS, CAMS/PUMC (Beijing, China) and cultured in Dulbecco’s Modified Eagle’s medium DMEM (DMEM, Gibco, Grand Island, NY, USA) containing 10% fetal bovine serum (FBS) (Gibco, Grand Island, NY, USA), 1% non-essential amino acids (NEAA) (Life technologies, Carlsbad, CA, USA) and 1% penicillin–streptomycin at 37 °C, 5% (v/v) CO2. HepG2 cells were serum-starved for 18 h and then treated with liraglutide (Novo Nordisk, Bagsværd, Denmark) at various concentrations (0, 10, 50, 100, 500 and 1000 nM) for 24 h or with 500 nM liraglutide for different times (0, 3, 6, 12, 24 h).

### Cell viability assay

The cell viability was determined using a Cell Counting Kit-8 (CCK-8; Dojindo Laboratories, Kumamoto, Japan) as previously described [24, 25]. Briefly, HepG2 cells were plated in 96-well plates (5 × 10^3^/well) and serum-starved for 18 h, and then exposed to different concentrations of liraglutide (0, 10, 50, 100, 500 and 1000 nM) for 24 h at 37 °C in a humidified atmosphere containing 5% CO_2_. After 24 h incubation, the CCK-8 solution was added to each well at a 1:10 dilution and incubated for an additional 3 h at 37 °C. The optical density (OD) value of each well was measured at a wavelength of 450 nm using a Microplate Reader (Thermo, Varioskan Flash). Cell viability was calculated by the following equation: viable cells (%) = (OD_test_ − OD_blank_)/(OD_control_ − OD_blank_). In the equation, OD_test_ is the optical density of the cells exposed to different concentrations of liraglutide, OD_blank_ is the optical density of the wells without HepG2 cells and OD_control_ is the optical density of the control sample (Phosphate-buffered saline, PBS). Results of cell viability assay are shown as the mean values of three replicate experiments performed in triplicate.

### Animal model and liraglutide treatment

Twenty-four male db/db mice (BKS.Cg-Dock7m +/+ Leprdb/J, Strain Number: J000642) aged 5 weeks and sixteen non-diabetic littermates (wild-type, WT) were purchased from the Model Animal Research Center of Nanjing University (Nanjing, China). All mice (four mice/cage) were housed under a 12-h light/dark cycle with 50% relative humidity at 22 °C and had free access to regular chow and water. After 2-week habituation, the mice were randomly divided into four treatment groups: db/db + LIRA (liraglutide) (n = 12), db/db + saline (n = 12), WT + LIRA (n = 8), WT + saline (n = 8). Mice were administered either liraglutide (200 μg/kg) or equivoluminal 0.9% saline subcutaneously, twice daily (09:00 and 18:00 h) for 7 weeks. Doses of the liraglutide used in the study were based on previous studies [26, 27]. During this period, body weight and 4-h fasting blood glucose levels from the tail vein were determined weekly. At the end of 7-week treatment period, the mice were euthanized after a 4-h fast and livers were removed after collecting blood for analysis. All experiments were approved by the Ethics Committee for Animal Care and Research at FuWai hospital (Beijing, China).

### Serum PCSK9 and serum lipid analysis

Mouse serum PCSK9 levels were detected by enzyme-linked immunosorbent assay (ELISA) using the CircuLex mouse/rat PCSK9 ELISA Kit (MBL, Nagano, Japan) according to the manufacturer’s instructions as previously described [9, 59]. The serum was diluted with 0.9% saline by 1:1. Then the levels of triglyceride (TG), total cholesterol (TC), LDL-C and high-density lipoprotein-cholesterol (HDL-C) were examined by the automatic biochemistry analyser (Beckman CX5, Beckman coulter, Brea, CA, USA).

### Hematoxylin and eosin (H&E) staining

Mouse liver slices were processed according to a standard a standard H&E staining technique [28]. Briefly, liver tissues were fixed by 10% neutral formalin, dehydrated in ethanol, and then embedded in paraffin. Liver sections (4 μm) were stained with hematoxylin and eosin (H&E).

### Oil red O staining

Oil red O staining was performed as previously described [29]. Briefly, mouse liver tissues were immediately snap-frozen in liquid nitrogen and placed in OCT cryostat embedding compound (Tissue-Tek, Torrance, CA, USA). Frozen liver sections (8 μm) were stained with Oil Red O, washed with 60% isopropanol, and counterstained with hematoxylin. Staining was assessed by bright-field microscopy.

### Immunohistochemistry and immunofluorescence

Mouse liver tissues were fixed with formaldehyde, embedded with paraffin and then cut into 4 μm-thick sections. Prior to immunostaining, the sections were dewaxed in xylene and rehydrated through graded alcohol. Antigen retrieval was carried out by boiling in citrate buffer (pH 6.0) for 2 min in a pressure cooker and then the cooker was depressurized and cooled under running water for 20 min [30]. Hydrogen peroxide (3%) was added to the tissue sections and incubated at room temperature for 10 min. Subsequently, the sections were washed with Phosphate buffered saline (PBS) for three times, and then incubated overnight with rabbit polyclonal PCSK9 antibody (1:200, Abcam, ab31762) or rabbit monoclonal LDLR antibody (1:500, Abcam, ab52818) at 4 °C. After that, the slides were incubated with goat anti-rabbit IgG/horseradish peroxidase (HRP) secondary antibody (Beijing Zhongshanjinqiao Biological Technology Co., LTD., China), and then counterstained with hematoxylin.

For immunofluorescence, the slides were blocked with 10% goat serum (Invitrogen, CA, USA) for 1 h and then incubated overnight with rabbit polyclonal PCSK9 antibody (1:200, Abcam, ab31762) or rabbit monoclonal LDLR antibody (1:500, Abcam, ab52818) at 4 °C. Goat anti-rabbit H&G (Alexxa Fluor 488, Abcam, ab150077) antibody was applied as the second antibody. 4′,6-diamidine-2′-phenylindole dihydrochloride (DAPI) (Beijing Zhongshanjinqiao Biological Technology Co., LTD., China) was used as nuclear counterstain.

### Western blots

Mouse liver tissue and HepG2 cells samples were homogenized on ice in lysis buffer containing protease and phosphatase inhibitors (Beyotime, Shanghai, China). The homogenate was then centrifuged at 12,000*g* for 15 min and the supernatant was collected. Protein concentrations were determined using a BCA Protein Assay Kit (Beijing Kangwei Century Biotechnology Co., Ltd, Beijing, China). Subsequently, 30 μg of protein from individual samples was resolved by precast NuPAGE Novex 4–12% (w/v) Bis–Tris gels (Life technologies, Carlsbad, CA, USA), and then transferred onto nitrocellulose membrane using the iBlotTM dry blotting system as described by the manufacturer (Invitrogen, Carlsbad, CA, USA). The membranes were blocked in TBST buffer (20 mM Tris, pH 7.5, 150 mM NaCl, 0.1% tween-20) containing 5% non-fat milk for 2 h at room temperature and then incubated overnight at 4 °C with anti-PCSK9 (1:1000, Abcam, ab31762), anti-HNF1α (Cell Signaling), anti-LDLR (1:5000, Abcam, ab52818) or anti-GAPDH (1:5000, Abcam). Afterwards, the membranes were incubated with the secondary antibodies including goat anti-rabbit IgG/horseradish peroxidase (HRP) and goat anti-mouse IgG/HRP (Abcam) for 2 h at room temperature. Protein expression was detected with chemiluminescence (ECL, Thermo Fisher Scientific, Waltham, MA, USA) on FluorChem M image system.

### Statistical analysis

Data are presented as mean ± standard error of the mean unless otherwise stated. Comparisons between two groups were assessed using an unpaired two-tailed Student *t* test. One-way ANOVA combined with Bonferroni’s post hoc test was used among ≥ 3 groups. Differences were considered statistically significant at P < 0.05. All analyses were performed using SPSS 19.0 (SPSS Inc., Chicago, IL, USA).

## Results

### Liraglutide down-regulated the protein expression of PCSK9 in HepG2 in a dose- and time-dependent manner

**Fig. 1:**
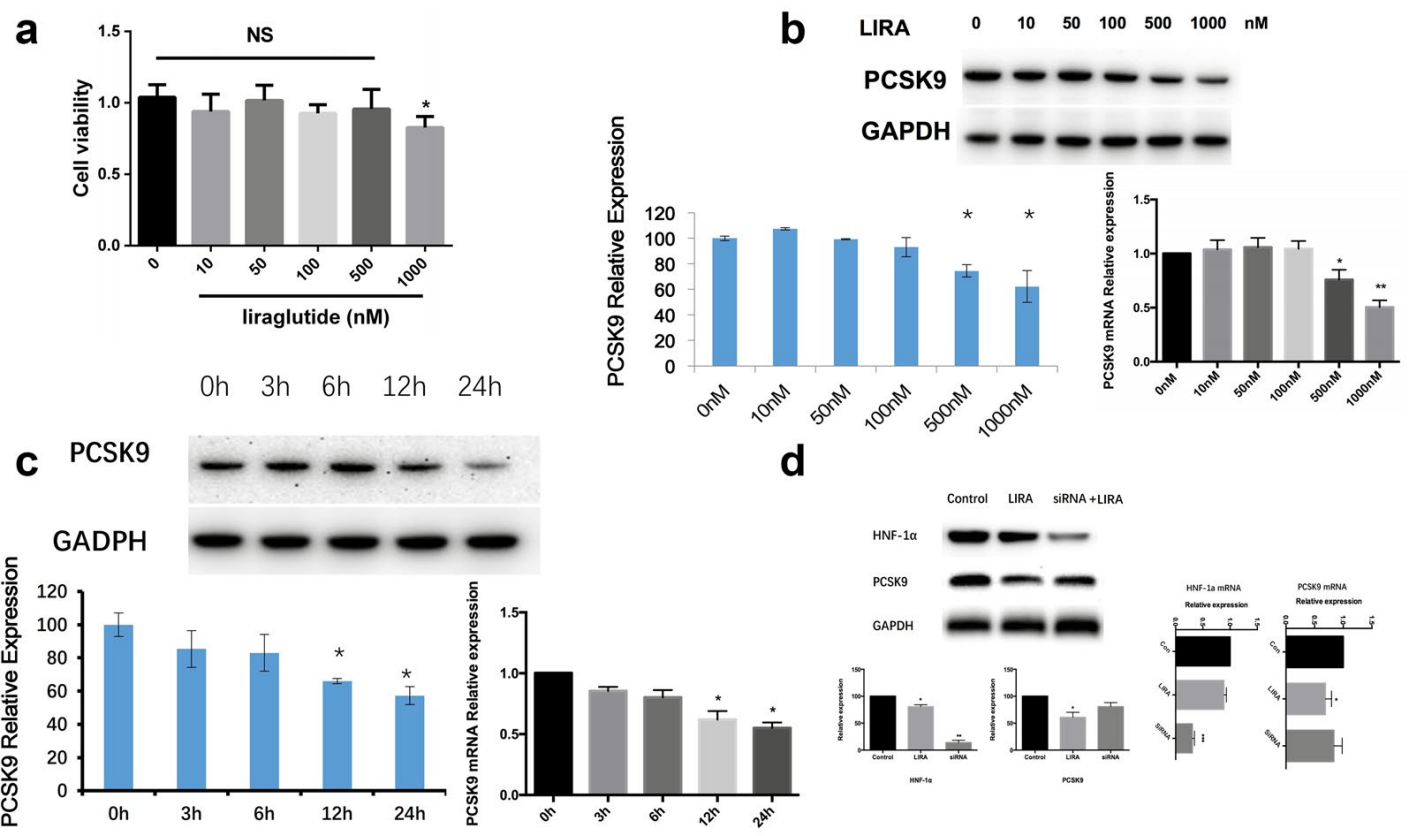
The effect of liraglutide on PCSK9 and HNF1α expressions in HepG2 cells. **a** HepG2 cells were incubated with different concentrations for 24 h and cell viability was determined by CCK-8 assay. **b** PCSK9 expression with liraglutide treatment in different concentrations (0, 10, 50, 100, 500, 1000 nM) for 24 h. **c** PCSK9 expression with liraglutide treatment in 500 nM for different times (0, 3, 6, 12, 24 h). **d** HNF1α expression with liraglutide treatment (500 nM) for 24 h. The normalized intensities of PCSK9 and HNF1α versus GAPDH are shown as mean ± SEM of three independent dose- and time-dependent experiments. *P < 0.05 vs. 0 nM liraglutide treatment (PBS treatment). ^#^P < 0.05. *NS* not significant, *Con* control, *LIRA* liraglutide

The HepG2 cells were treated with liraglutide (10, 50, 100, 500 and 1000 nM) for 24 h and their viabilities were assessed using the CCK-8 assay. As shown in Fig. 1a, liraglutide showed no cytotoxicity below 1000 nM (1 μM). Subsequently, we determined whether liraglutide could affect the expression of PCSK9 in HepG2 and found that liraglutide down-regulated the protein and mRNA levels of PCSK9 in a dose-dependent manner (Fig. 1b). In parallel, we also found that liraglutide had time-dependent inhibitory effect on the PCSK9 protein and mRNA expression in HepG2 (Fig. 1c). Also, the protein and mRNA expression of HNF1α was found to decrease when HepG2 cells were exposed to liraglutide (500 nM) for 24 h (Fig. 1d). Moreover, the inhibiting effect of liraglutide on PCSK9 was weakened after inhibition of HNF1α by siRNA (Fig. 1d).

### Liraglutide decreased body weight and improved glucose metabolism

**Fig. 2:**
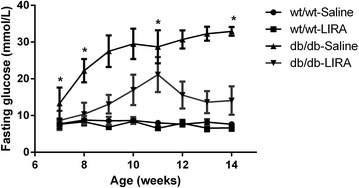
Change in body weight (**a**) and fasting glucose (**b**) in db/db mice and nondiabetic mice that were administered liraglutide for 7 weeks starting at age 7 weeks. *P < 0.05 versus control db/db or WT mice (n = 8–12 in each group). *WT-Saline* wild-type (nondiabetic) mice treated with saline, *WT-LIRA* wild-type (nondiabetic) mice treated with liraglutide, *db/db-Saline* db/db mice treated with saline, *db/db-LIRA* db/db mice treated with liraglutide

The db/db mice had higher levels of fasting blood glucose than those of the WT mice (Fig. 2). Seven-week old male mice (WT or db/db mice) were administered liraglutide (200 μg/kg, twice daily) or vehicle (saline) subcutaneously for 7 weeks. As expected, liraglutide treatment decreased body weight in both WT mice and db/db mice (data not shown) and significantly reduced blood glucose levels in db/db mice but not in WT mice (Fig. 2).

### Liraglutide reduced lipid accumulation in the serum and liver in db/db mice

**Fig. 3:**
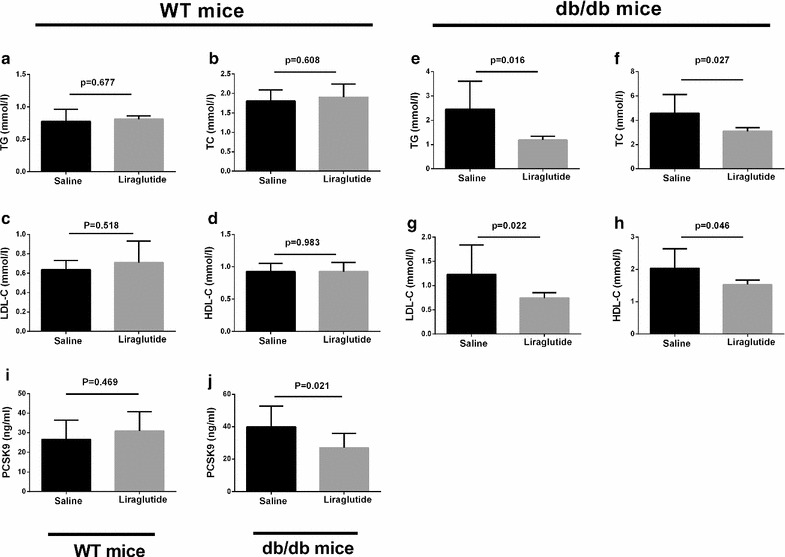
Effects of liraglutide on serum lipid profile and PCSK9 level in db/db mice and nondiabetic mice (n = 8–12 per group). **a**–**d** serum levels of TG, TC, LDL-C and HDL-C in WT mice; **e**–**h** serum levels of TG, TC, LDL-C and HDL-C in db/db mice; **i** serum PCSK9 level in WT mice; **j** serum PCSK9 level in db/db mice. *WT* wild-type

**Fig. 4:**
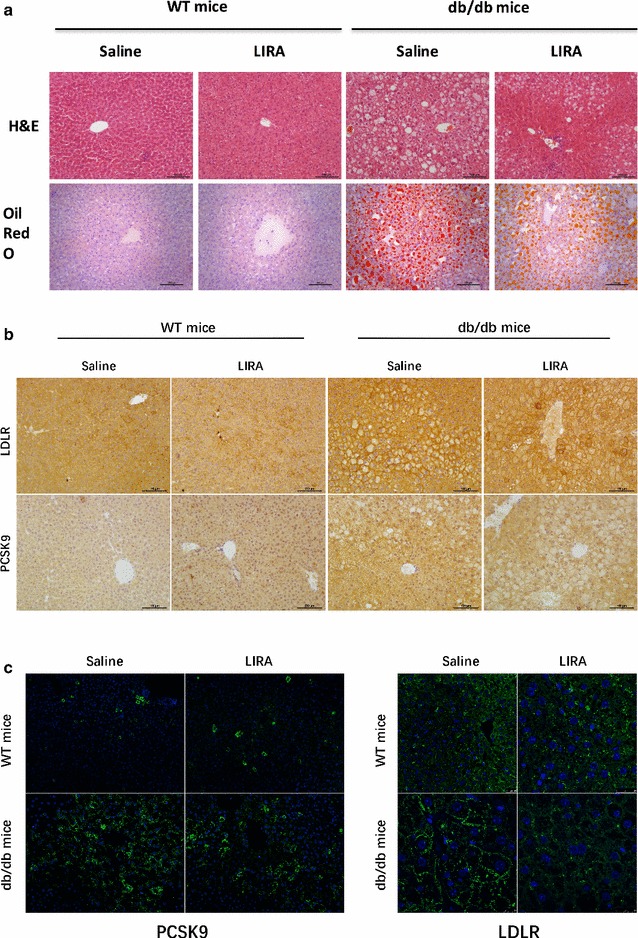
Effects of liraglutide on hepatic steatosis, hepatic PCSK9 and LDLR proteins by staining with H&E or Oil Red O (**a**), immunohistochemical detection (**b**) or immunofluorescence detection (**c**) in db/db and wild type mice. Magnification, ×200 (hematoxylin and eosin stain and oil red O). *LIRA* liraglutide

There were no differences in the levels of serum TC, TG, LDL-C and HDL-C among the non-diabetic WT mice with liraglutide or saline treatment (Fig. 3a). In contrast, the levels of TC (3.10 ± 0.10), TG (1.19 ± 0.05) and LDL-C (0.74 ± 0.03) in db/db mice that were treated with liraglutide were significantly decreased compared with those of control db/db mice (TC: 4.59 ± 0.54, P = 0.027; TG: 2.46 ± 0.40, P = 0.016; LDL-C: 1.23 ± 0.21, P = 0.022) (Fig. 3b) Interestingly, liraglutide also reduced the serum HDL-C level in db/db mice (Fig. 3b). Furthermore, oil red O staining of liver sections exhibited no apparent lipid deposition in the livers of non-diabetic WT mice regardless of the treatment of liraglutide (Fig. 4a). Conversely, in db/db mice, marked accumulation of oil red O-stainable lipid droplets was found in liver section and a significantly decrease in the number of the lipid droplets was shown in liraglutide-treated db/db mice compared with the vehicle-treated db/db mice (Fig. 4a).

### Liraglutide suppressed PCSK9 levels in db/db mice

**Fig. 5:**
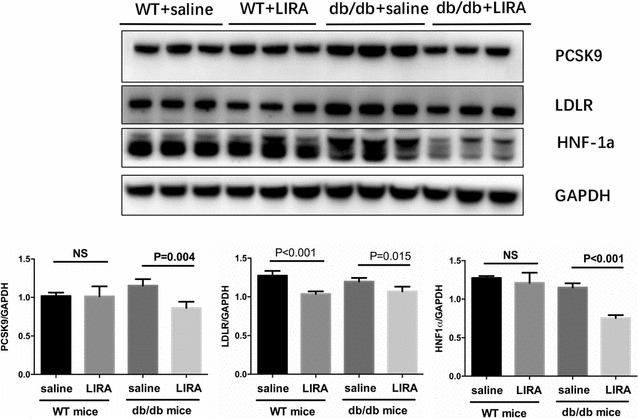
Effects of liraglutide on hepatic PCSK9, LDLR and HNF1α expressions in db/db mice and nondiabetic mice. *WT* wild type, *LIRA* liraglutide

To determine whether liraglutide can affects PCSK9 levels, we detected the serum and liver PCSK9 levels in WT and db/db mice. After liraglutide treatment for 7 weeks, there was no difference in serum PCSK9 level in WT mice compared with saline-treated mice (26.57 ± 3.51 vs. 30.82 ± 4.45, P = 0.464), whereas the serum level of PCSK9 in db/db mice exhibited a marked decrease (39.93 ± 4.50 vs. 26.97 ± 2.78, P = 0.021) (Fig. 3i, j). Meanwhile, immunohistochemistry and immunofluorescence staining revealed that liraglutide decreased liver PCSK9 protein level in db/db mice but not in WT mice (Fig. 4b, c), which is consistent with the western blots results (Fig. 5). In parallel, we assessed the liver LDLR level in db/db and WT mice and unexpectedly found that liraglutide reduced the LDLR level in both db/db mice and WT mice (Figs. 4c, 5). Importantly, liraglutide showed an inhibitory effect on the hepatic HNF1α expression in db/db mice not in WT mice (Fig. 5).

## Discussion

Based on the data regarding the relation of PCSK9 to cholesterol and glucose metabolism and the beneficial effects of liraglutide on cardiovascular health, we set out to explore whether liraglutide has an effect on PCSK9 expression. Firstly, data showed that liraglutide down-regulated PCSK9 expression in HepG2 cells in a dose- and time-dependent fashion through HNF1α-dependent mechanism. In addition, the results indicated that liraglutide could reduce serum and hepatic PCSK9 levels in db/db mice rather than in WT mice.

Liraglutide, a long-acting GLP-1 receptor agonist, is widely used for treating diabetes by stimulating glucose-dependent insulin secretion and suppressing glucagon secretion with a very low risk for hypoglycemia [19, 31, 32]. In addition, liraglutide can induce body weight loss through reducing food intake, promoting satiety, delaying gastric emptying and inducing autophagy [21, 33–35]. Similarly, our data showed that liraglutide lowered serum glucose levels in db/db mice. In fact, previous study suggested that liraglutide could markedly modify circulating lipid profile levels in patients with type 2 diabetes or in diabetic mice [19–21, 36], which was also demonstrated by our study. The mechanisms of liraglutide on lipid profile have been speculated: (1) to increase insulin secretion and to reduce eating [37, 38]; (2) to reduce chylomicron production, lipoprotein synthase and ApoB-48 production [39]; (3) to slow gastric emptying and to reduce lipase activity [40]; (4) to decrease VLDL production, lipogenesis and ApoB100 production [41, 42]; (5) to inhibit fat oxidation and to promote thermogenesis [43]; (6) the effect of decreased PCSK9 by liraglutide on cholesteryl ester transfer protein (CETP) inhibition on lipid profiles [44]. Additionally, we observed that liraglutide significantly improved hepatic lipid accumulation in db/db mice, which is consistent with some researches [45–48]. Conversely, some other studies showed that liraglutide treatment did not reduce hepatic steatosis [49, 50]. All these inconsistent results may be attributed to the various definitions, evaluation methods, doses or durations of liraglutide treatment.

A novel finding of the present study is that liraglutide can inhibit PCSK9 expression at the cellular and animal levels, which may be an explanation for the beneficial effects of liraglutide on cardiovascular outcomes. Moreover, we found that liraglutide also reduced HNF1α expression. To explore whether HNF1α, a critical regulator of PCSK9 gene transcription, is involved in the regulation of PCSK9 expression, we inhibited HNF1α expression using siRNA in HepG2 cells. As expected, the data showed that the inhibiting effect of liraglutide on PCSK9 was weakened after inhibition of HNF-1α by siRNA, suggesting that liraglutide reduced PCSK9 at least partly via HNF1α-dependent mechanism. Meanwhile, we found that SREBP-2 was not involved in the effect of liraglutide on PCSK9 (data not shown), which is supported by our previous studies [51, 52]. Also, in the present study, liraglutide reduced PCSK9 expression in HepG2 and db/db mice but not in WT mice. This is perhaps due to the fact that pathological states may alter the role of liraglutide in PCSK9 expression, but its real reason is unknown.

Unexpectedly, decreased levels of hepatic PCSK9 protein were not associated with an increase in hepatic LDLR protein in db/db mice, namely, liraglutide also inhibit LDLR. Actually, previous studies reported this phenomenon [53–55]. Miao et al. showed that insulin promoted the degradation of LDLR in a PCSK9-dependent manner in HepG2 cells, but in vivo in insulin-deficient states, both PCSK9 and LDLR levels reduced [53]. Similarly, Levenson et al. reported that leptin suppressed both PCSK9 and LDLR in *ob/ob* mice [54]. Moreover, liraglutide reduced both hepatic PCSK9 and LDLR expression simultaneously, and it is possible that another regulator such as inducible degrader of LDLR (Idol) promotes degradation of LDLR [56]. Moreover, glucagon has been demonstrated to increase LDLR [57]. Liraglutide can suppress glucagon secretion [58] and thus may contribute to LDLR reduction. However, the real season remains unclear. All these data point to the fact that the role of some reagents such insulin, leptin and liraglutide and so on in LDLR regulation is complex, and suggests that in vivo these reagents may act through PCSK9-independent mechanism to affect LDLR expression. Nevertheless, further researches are needed to confirm our finding.

This study has several limitations. Firstly, the db/db model used in the study was characterized by hyperinsulinemia, so we could not find out whether insulin affected LDLR expression. Even so, this model was also used in previous to explore the effect of PCSK9 on glucose and lipid metabolism [59]. Secondly, the mechanism of the effect of liraglutide on LDLR was not explored. Finally, we failed to find out whether liraglutide had a major role in lipid metabolism compared to insulin due to the animal model we used.

## Conclusion

In conclusion, our data indicated that liraglutide suppressed PCSK9 in HepG2 cells and db/db mice through HNF1α-dependent mechanism, and decreased LDLR possibly via PCSK9-independent pathways in db/db mice (Additional files 1, 2, 3, 4).

## Additional files

**Additional file 1.** Change in fasting glucose in db/db mice and nondiabetic mice.

**Additional file 2.** Effects of liraglutide on hepatic steatosis by staining with H&E or Oil Red O.

**Additional file 3.** Immunohistochemical detection of heptatic PCSK9 and LDLR proteins.

**Additional file 4.** Immunofluorescence detection of heptatic PCSK9 and LDLR proteins.

## Abbreviations

GLP-1: glucagon-like peptide-1
HepG2: human hepatoma cell
HNF-1α: hepatocyte nuclear factor 1 alpha
HDL-C: high-density lipoprotein-cholesterol
LDLR: low-density lipoprotein receptor
LDL-C: low-density lipoprotein cholesterol
LIRA: liraglutide
TC: total cholesterol
TG: triglyceride
PCSK9: proprotein convertase subtilisin/kexin type 9
WT: wild type
