# Supplementary figures and images for: Liraglutide downregulates hepatic LDL receptor and PCSK9 expression in HepG2 cells and db/db mice through a HNF-1a dependent mechanism
Source: Cardiovasc Diabetol. 2018 Apr 4;17:48. doi: 10.1186/s12933-018-0689-9 (PMC5885408; doi:10.1186/s12933-018-0689-9)

## Slide 1
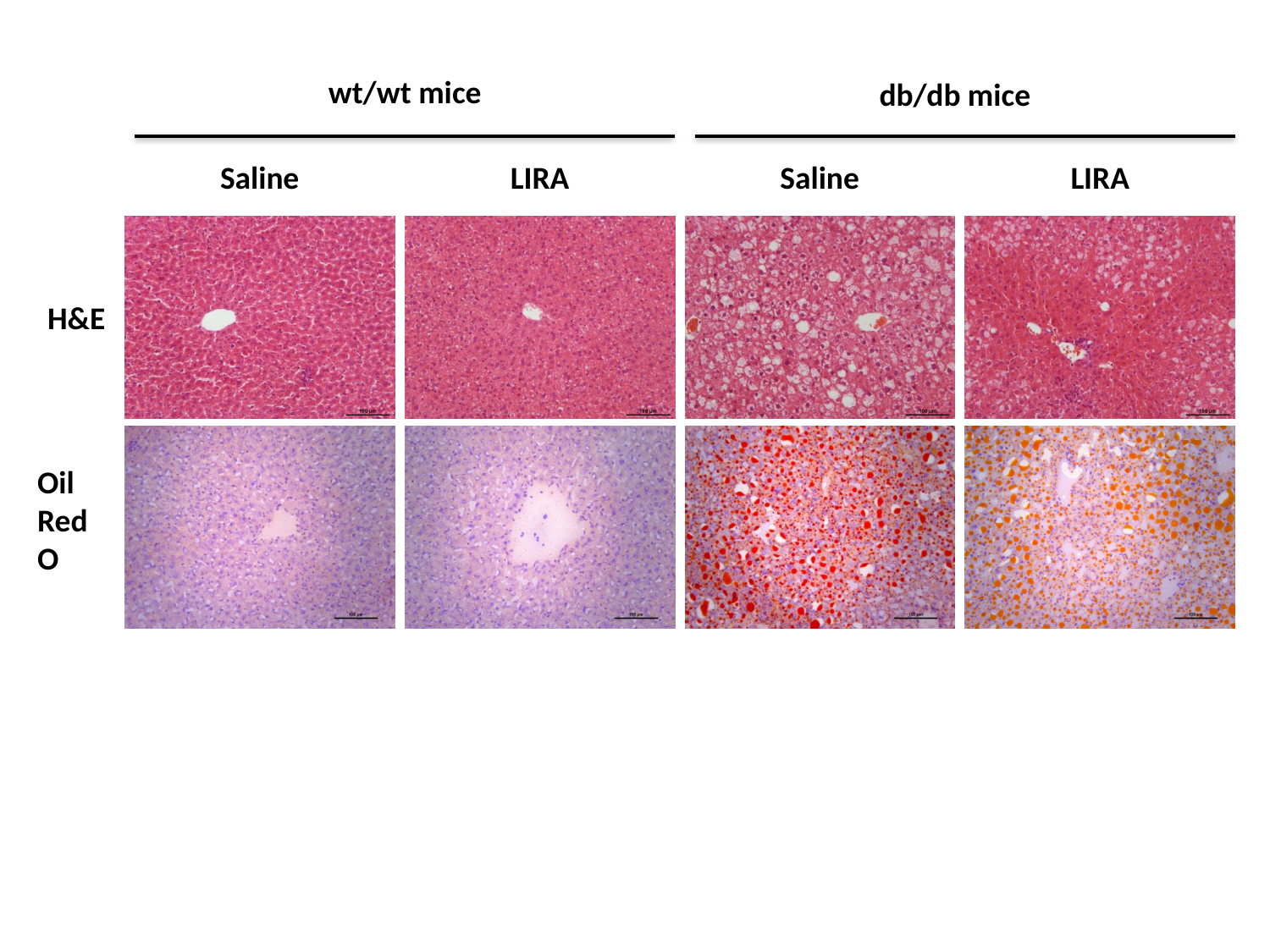

wt/wt mice
db/db mice
Saline
LIRA
Saline
LIRA
H&E
Oil Red O

Supplement: Supplementary file 2 — Additional file 2. Effects of liraglutide on hepatic steatosis by staining with H&E or Oil Red O. [file 12933_2018_689_MOESM2_ESM.pptx]

## Slide 1
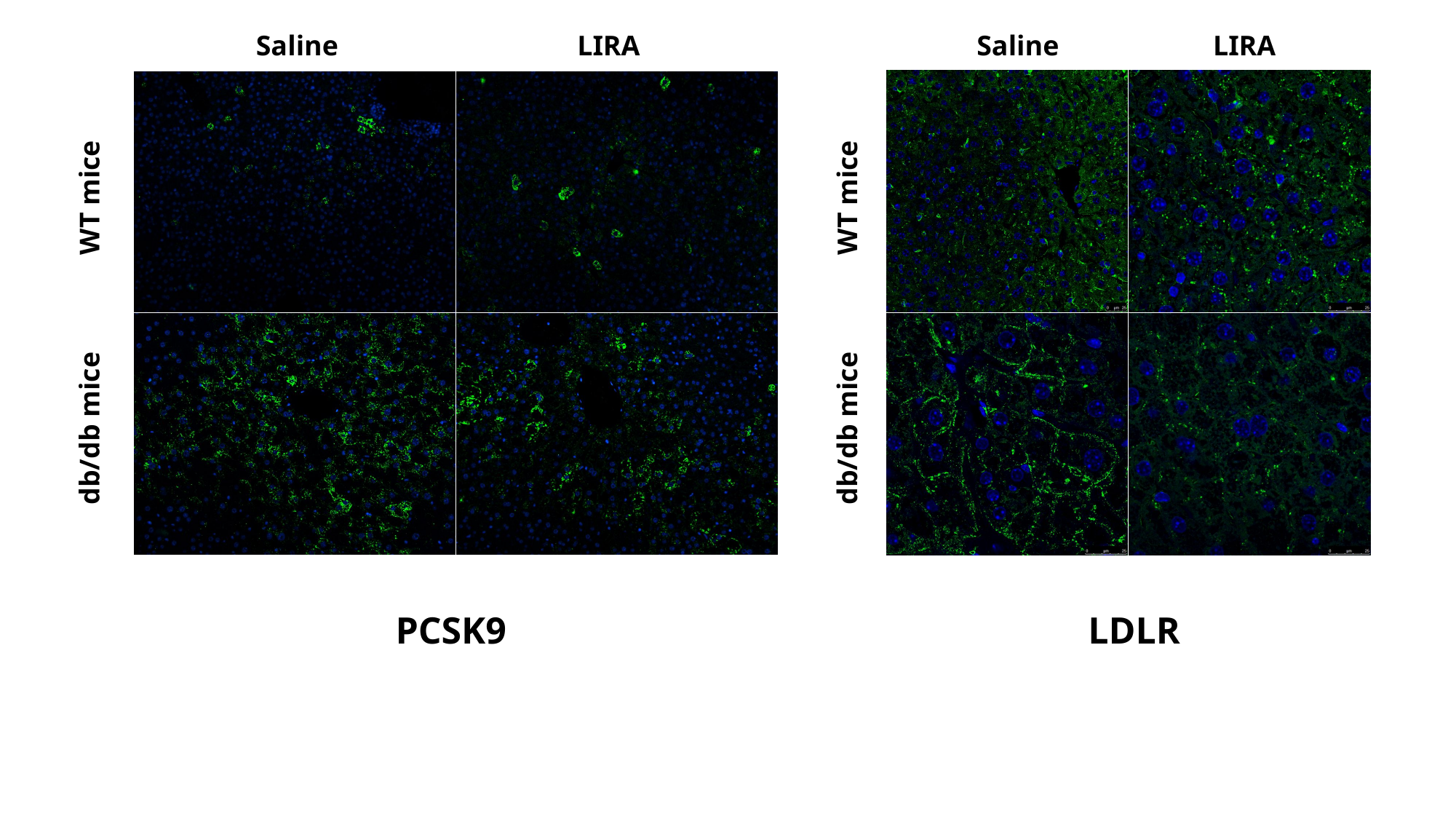

Saline
LIRA
Saline
LIRA
WT mice
WT mice
db/db mice
db/db mice
 LDLR
 PCSK9

Supplement: Supplementary file 4 — Additional file 4. Immunofluorescence detection of heptatic PCSK9 and LDLR proteins. [file 12933_2018_689_MOESM4_ESM.pptx]
